# Peripheral Exudative Hemorrhagic Chorioretinopathy (PEHCR): Diagnostic and Therapeutic Challenges

**DOI:** 10.3390/medicina59091507

**Published:** 2023-08-22

**Authors:** Kevin F. Elwood, Paige J. Richards, Kathleen R. Schildroth, Mihai Mititelu

**Affiliations:** Department of Ophthalmology and Visual Sciences, University of Wisconsin-Madison School of Medicine and Public Health, Madison, WI 53705, USA

**Keywords:** choroidal neovascular membrane, pseudomelanoma, PEHCR, retinal vascular peripheral lesion, anti-vascular endothelial growth factor (anti-VEGF)

## Abstract

*Background and Objectives:* Peripheral exudative hemorrhagic chorioretinopathy (PEHCR) is a peripheral retinal vascular abnormality that is likely underreported. We review the differential diagnoses, etiology, and treatment options for PEHCR. *Methods:* We present a case of an asymptomatic 72-year-old female referred following left eye fundus photography finding of the peripheral lesion. *Results:* Fundus photography demonstrated a large temporal pigment epithelial detachment (PED) with adjacent fibrovascular membrane. Optical coherence tomography (OCT) confirmed the PED with trace subretinal fluid. Fluorescein angiography (FA) demonstrated early and late hypofluorescence of the PED with late leakage of the adjacent temporal fibrovascular membrane. Observation was elected, visual acuity remained unaffected, and the PED spontaneously resolved. *Conclusions:* Due to the peripheral location, patients often present as asymptomatic; however, vision loss can occur due to vitreous hemorrhage or extension of subretinal fluid, hemorrhage, or exudate to the macula. Commonly, these lesions are referred with concern for choroidal melanoma due to their large, dark, elevated presentation in the peripheral retina. Multimodal testing using B-scan, FA, and OCT is important in establishing the proper diagnosis. PEHCR lesions can often be observed without treatment, though intravitreal injection of anti-VEGF is increasingly used to prevent secondary causes of vision loss.

## 1. Introduction

Peripheral exudative hemorrhagic chorioretinopathy (PEHCR) is a degenerative disease of the peripheral retina. This condition was first reported by Reese and Jones in 1961, though first named by Annesley in 1980 [1,2]. It commonly presents asymptomatically in older individuals, although it is associated with subretinal hemorrhage, sub-retinal pigment epithelium (RPE) hemorrhage, subretinal fluid (SRF), and exudation [3,4]. Most cases are self-limiting, but vision loss has been reported from subretinal hemorrhage or exudation extending from the periphery towards the macula, as well as breakthrough vitreous hemorrhage, which is a significant cause of vision loss [5]. PEHCR has been named a variant of age-related macular degeneration (AMD) by some due to the similar pathology [3]. Additionally, PEHCR has been described to be a variant of polypoidal choroidal vasculopathy (PCV) based on their common highly hemorrhagic and exudative characteristics, in addition to some cases having polypoidal lesions seen on indocyanine green angiography (ICGA) [6].

PEHCR can commonly be mistaken for choroidal melanoma, retinal detachment, posterior vitreous degeneration, or retinal artery macroaneurysm [2]. With its often dark and elevated appearance in the peripheral retina, it is the second most common diagnosis of ‘pseudomelanoma’ at 13%, in addition to other differential diagnoses such as choroidal nevus (49%), congenital hypertrophy of the RPE (6%), circumscribed choroidal hemangioma (5%), and AMD (4%) [4,7]. Additionally, the exudative type of PEHCR can mimic primary vitreoretinal lymphoma (PVRL) [4].

Given the sight- and potentially life-threatening conditions that PEHCR mimics, it is important to properly recognize and diagnose this condition. This can avoid unnecessary invasive testing and guide treatment when indicated.

## 2. Clinical Case

**Figure 1:**
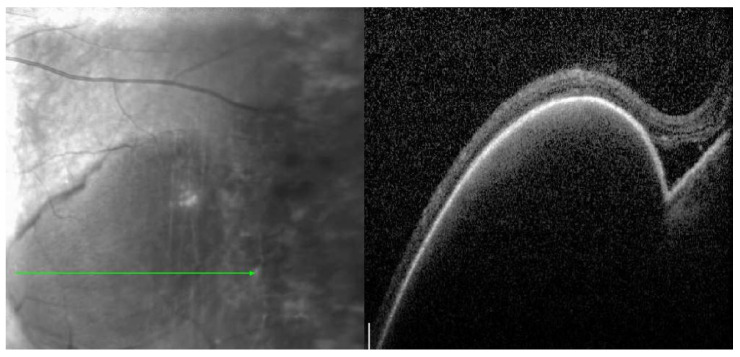
**Red-free image with green arrow corresponding to** SD-OCT of the left eye demonstrating large pigment epithelial detachment (PED) temporal to the macula with a small amount of the subretinal fluid.

**Figure 2:**
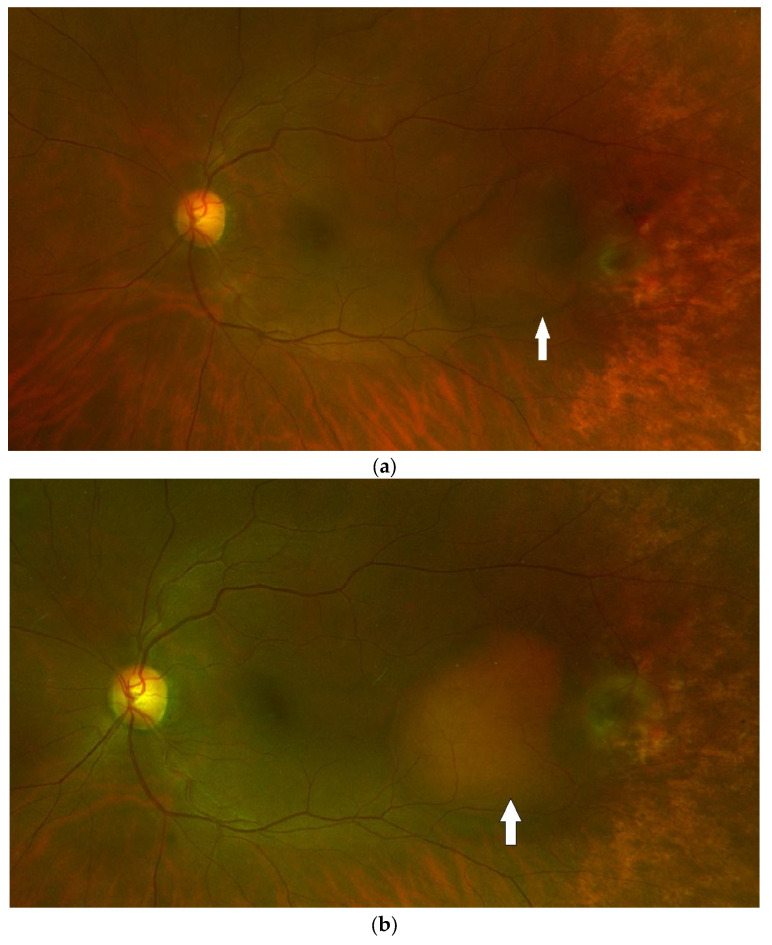
Fundus photography of the left eye. (**a**) On presentation, a large temporal PED (indicated by white arrow) with adjacent grey fibrovascular membrane temporally and small amount of subretinal hemorrhage superiorly is demonstrated. Peripheral retinal degeneration is appreciated in the temporal periphery; (**b**) Large temporal PED (indicated by white arrow) is stable with resolved hemorrhage 3 months later.

**Figure 3:**
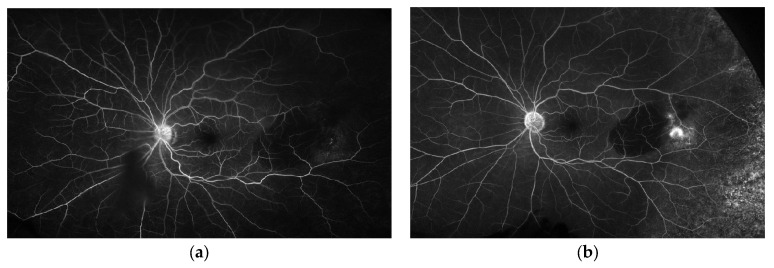
Fluorescein angiogram of the left eye. (**a**) Early phase hypofluorescence of the temporal PED; (**b**) Late phase hypofluorescence of the temporal PED with hyperfluorescent leakage of the lesion temporal to the PED.

**Figure 4:**
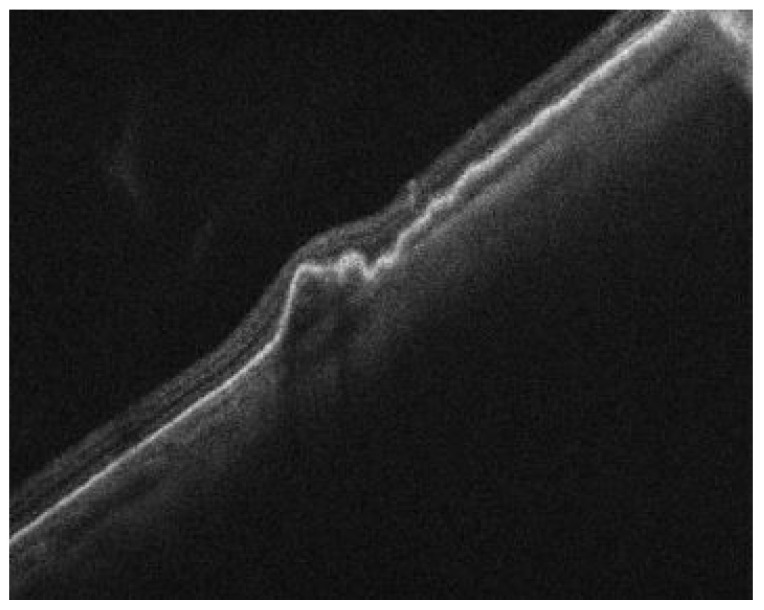
SD-OCT of the left eye demonstrating nearly resolved pigment epithelial detachment (PED) temporal to the macula with no subretinal fluid.

A 72-year-old woman with a past medical history of hypertension and age-related cataracts presented to the retina clinic for evaluation of an asymptomatic choroidal lesion in the left eye. The lesion was discovered by fundus photography during a routine eye examination by her optometrist with concern for new, large, temporal pigmentary changes of unknown etiology. Her visual acuity (VA) was 20/25 in the right eye and 20/30 in the left eye. Her ocular examination was significant for an elevated and grey temporal chorioretinal lesion with a small amount of adjacent old subretinal hemorrhage. Peripheral spectral-domain optical coherence tomography (SD-OCT) (Spectralis, Heidelberg Engineering, Inc., Heidelberg, Germany) of the left eye showed a pigment epithelial detachment (PED) temporal to the macula with a small amount of subretinal fluid (Figure 1). This PED was suspected to be hemorrhagic based on the heterogenous hyporeflective appearance and concordance with supporting imaging modalities. The subretinal fluid was located overlying the old subretinal hemorrhage seen on fundoscopic examination. Ultra-widefield color fundus photography (Optos, Inc., Marlborough, MA, USA) of the left eye showed a large PED temporal to the macula with adjacent hemorrhage and a grey fibrovascular membrane temporal to the PED (Figure 2). B-scan ultrasonography demonstrated a temporal hypoechoic elevated lesion with a height measuring 1.54 mm and a base of 6.76 mm × 7.30 mm. Fluorescein angiography (FA) showed early and late hypofluorescence of the temporal PED and late hyperfluorescent leakage of the lesion temporal to the PED (Figure 3). This blocking pattern was consistent with the suspected hemorrhage. A diagnosis of peripheral exudative hemorrhagic chorioretinopathy (PEHCR) was made. The option of treatment with an intravitreal injection of bevacizumab was discussed, but ultimately the patient chose observation without treatment given she was asymptomatic and had good VA. The lesion appeared stable clinically at a 3-month follow up (Figure 2b) with resolved hemorrhage, and B-scan measurements also remained stable. At follow-up 6 months later, the PED had nearly resolved (Figure 4), and the patient remained symptom-free having not required treatment such as an anti-vascular endothelial growth factor (VEGF) injection.

## 3. Discussion

We report a case of PEHCR in an asymptomatic 72-year-old female who deferred treatment due to unaffected vision and peripheral lesion location. While PEHCR most commonly does not cause vision decline, it can result in vision loss secondary to vitreous hemorrhage or macular extension of hemorrhage, subretinal fluid, or exudate [4]. Shields et al. in particular report 21% of eyes present with decreased vision on presentation: 14% due to vitreous hemorrhage, 5% due to subretinal hemorrhage, and 2% due to subretinal fluid in their population [4]. Zicarelli et al. hypothesize that peripheral subretinal fluid and the presence of diffuse or subtotal PEHCR lesions are the main risk factors for visual impairment due to macular involvement or vitreous hemorrhage [8].

The vast majority of patients with PEHCR are Caucasian [4,8,9,10]. Shields et al. report that out of 146 patients, 99% of this cohort were Caucasian and 1% (2 patients) were of Asian ethnicity [4]. Within this same cohort, 67% of patients were female, and the overall mean age was 80 years of age, ranging from 57 to 97 [4]. In a separate large case review by Mantel et al., 100% of the 46 patients with PEHCR were white, 69% female, and there was a mean age of 77 with a range from 60 to 91 [9]. In this cohort, only 6.5% of patients were on anticoagulation treatment [9]. Some 44% of the Shields et al. cohort were on anticoagulation medication [4]. In terms of previous eye treatment, 88% had no prior treatment, 7% had prior pars plana vitrectomy for vitreous hemorrhage, 3% had laser photocoagulation for age-related macular degeneration, 2% had photodynamic therapy for age-related macular degeneration, and 1% had macular translocation for age-related macular degeneration [4]. 

While PEHCR is often asymptomatic, it can present with decreased vision, flashes or floaters, and, rarely, pain [4]. Though the most common presentation was asymptomatic in the Shields cohort, Vandefonteyne et al. reported on a cohort of 84 eyes, 69 patients, of whom 83.3% presented with symptoms [10]. This included vision loss in 80.7%, floaters in 31.1%, flashes in 13.6%, metamorphopsia in 11.1%, scotoma in 8.9%, visual field defect in 8.9% and pain in 2.1% [10]. PEHCR can present as both a unilateral or bilateral disease process, and reports in the literature have found a 30% bilateral rate of involvement at the time of diagnosis [4]. The most common lesion location is in the temporal quadrant, more specifically inferotemporally [10]. 

PEHCR is critical to consider in the differential diagnosis for an elevated dark lesion in the periphery and can masquerade serious conditions. PEHCR is the second leading pseudomelanoma, following choroidal nevus [4]. In the Shields et al. cohort, the referring diagnosis was choroidal melanoma in 99% of patients [4]. Of the 56 eyes referred to Mantel et al., the referring diagnosis was choroidal neoplasm in 87% (48% for malignant melanoma, 4.4% for choroidal metastasis, and 33.3% for tumor of unknown origin) [9]. Other referring diagnoses included retinal schisis, sclerochoroidal calcification, hemorrhagic glaucoma, pigment epithelium detachment, and unknown [9]. The condition is classified into two subtypes: the hemorrhagic type greatly resembles malignant melanoma, while the exudative type resembles primary vitreoretinal lymphoma (PVRL) [11]. In contrast to choroidal melanoma and PVRL, PEHCR generally carries a better prognosis [10]. The mean diameter of the PEHCR lesions were 10 mm with a mean thickness of 3 mm [4]. A total of 77% of the lesions were located temporally and 89% located between the equator and the ora serrata [4]. Additional features of PEHCR include subretinal hemorrhage, retinal exudation, RPE detachment, and sub-RPE hemorrhage. Some 42% of eyes had drusen or peripheral RPE alterations in the fellow eye [4]. Macular drusen, choroidal neovascularization, or RPE changes were found in 48% of ipsilateral eyes and 56% of contralateral eyes [4]. With a mean observation time of 15 months, 89% of lesions stabilized or regressed while only 11% progressed [4]. 

The pathophysiology of PEHCR is not fully understood. PEHCR has been thought of by some to be a variant of AMD due to their similar demographic population and common signs such as accompanying RPE changes, drusen, and choroidal neovascularization. However, fluorescein angiography and pathologic evaluation of enucleated eyes with PEHCR found a choroidal vascular network only in a few cases [10]. The recognition of accompanying macular changes in some cases of PEHCR may be due to the advanced age at presentation and therefore may represent completely separate clinical entities. 

PEHCR presents with degenerative changes in the retina and RPE, of which hemorrhagic RPE detachments are frequent manifestations, as highlighted in our case in this report. Mashayekhi et al. report an interesting case of PEHCR in which smaller peripheral hemorrhagic PEDs gradually enlarged and coalesced into a large hemorrhagic PED [12]. The peripheral choroidal vascular changes seen on ICGA resembled polypoidal choroidal vasculopathy (PCV) corresponding to the location of the PEDs. This led the authors to hypothesize that PCV may underlie the development of PEHCR in some cases [12]. PCV classically presents as a reddish-orange subretinal nodule [13]. Both conditions are typically associated with serous and hemorrhagic PED, subretinal hemorrhage, lipid exudation, and a remitting–relapsing course with recurrent hemorrhagic episodes [12]. It is important to distinguish that PCV is typically macular or peripapillary in location while PEHCR is by definition peripheral. In addition, PCV is more prevalent in Asian and African Americans than Caucasians and also has a slightly younger mean age of patients (60–72 years) than PEHCR [13]. While PCV and PEHCR may represent different clinical manifestations of the same disease, Mashayekhi et al. note that these diseases may simply share a similar underlying vascular pathology and may in fact be separate entities as opposed to related etiologies on the same spectrum [12]. 

Multimodal imaging can help avoid misdiagnosis of PEHCR and guide treatment and disease monitoring. Vandefonteyne et al. report multimodal imaging findings of PEHCR, with fluorescein angiography (FA) showing CNV in 26.3% of eyes and peripheral diffusion in 60.5% of cases [10]. Additionally, ICG testing demonstrated polyps in 58.3% of eyes, while SD-OCT showed a wide range of macular findings: subretinal fluid (12.5%), macular edema (18.8%), PED (6.3%), CNV (4.2%), macular fibrosis (8.3%), and epiretinal membrane (25%) [10]. In recent years, OCT has been increasingly used to evaluate peripheral vitreoretinal pathologies such as retinal detachments, retinal holes, retinal tears, retinoschisis, and retinal tufts [14]. The use of peripheral OCT was instrumental in the evaluation and diagnosis of our case discussed above, allowing for clear visualization of the PED and small area of subretinal fluid. The ability to monitor disease progression with peripheral OCT can help determine indication for potential treatment. B-scan echogenicity was hollow (8.8%), intermediary (8.8%), or solid (23.5%), with most cases demonstrating a heterogeneous appearance (58.8%) [10]. Ultra-widefield imaging is a helpful clinical tool to aid in the diagnosis and monitoring of PEHCR given the peripheral location of these lesions, often anterior to the equator. Widefield imaging, including widefield angiography, has been well documented as a reliable method of diagnosis and monitoring of PEHCR lesions [7,8,9]. An additional comprehensive review of 35 eyes found the most common OCT and ultrasound findings to be a subretinal mass (68.75%), PED (30%), and atrophic changes (21.86%) [11]. On ultrasound, most eyes (88.46%) showed moderate to high internal reflectivity. Of the seven eyes with fundus autofluorescence, all demonstrated both high and low autofluorescence of the lesion [11]. FA was available in nine eyes with five eyes demonstrating lesion hyperfluorescence, of which two eyes revealed leakage, two revealed staining, and one revealed pooling [11].

Zicarelli et al. recently published a series of 50 eyes of 35 patients with PEHCR with extensive multimodal image results. In this cohort, the mean age was 83 years, 74% of patients were female, 100% were Caucasian, the mean best corrected visual acuity at presentation was 20/40, the mean best corrected visual acuity at final visit was 20/40, 60% of patients were asymptomatic, 57% unilateral while 43% had bilateral lesions, with a referring diagnosis of choroidal melanoma in 38% and bleeding/exudation in 62% [8]. At presentation, the PEHCR lesion was focal (defined as lesion for 3 or fewer clock hours) in 22 eyes (44%), diffuse (defined as lesion for 4 to 6 clock hours) in 17 eyes (34%), and a subtotal (defined as greater than 6 clock hours) in 11 eyes (22%) [8]. PEDs were seen in 100% of eyes [8]. Subretinal fluid adjacent to the peripheral lesions was in more than half of the cases (62%) and subretinal/sub-RPE blood in 35 eyes (70%) [8]. ICGA revealed peripheral polypoidal lesions in 27 eyes (54%), peripheral leakage in 31 eyes (62%), and peripheral vascular networks in 42 eyes (84%) [8], though the authors note that neither the ICGA findings of leakage nor polypoidal dilation led to worse outcomes [8]. In this cohort, macular involvement was noted in 17 eyes (34%) and intravitreal blood was noted in 7 eyes (14%) [8]. PEHCR lesions with peripheral extension greater than 3 clock hours statistically significantly had more macular involvement [8]. PEHCR extension also statistically significantly correlated with intravitreal blood [8]. Interestingly, Zicarelli et al. found that subretinal or sub-RPE bleeding did not correlate with macular involvement or vitreous hemorrhage [8]. This suggested to the authors that the classic peripheral hemorrhage characterizing PEHCR lesions may be self-limiting and visually insignificant. 

Anti-vascular endothelial growth factor (VEGF) injections, cryotherapy, and laser photoablation have been proposed treatment modalities, though no statistically significant impact on any method has been observed [11]. Most PEHCR is self-limiting and lesions spontaneously resolve and do not require treatment [4]. RPE atrophy, hyperplasia, and fibrosis are commonly seen following lesion regression [4]. An anti-VEGF intravitreal injection appears to be the most commonly utilized treatment to stop lesion progression, especially in cases with macular extension of subretinal fluid, hemorrhage, or exudate. Multiple rounds of anti-VEGF injections have been utilized for persistent or recurrent lesions [11]. In a larger case series following widespread adoption of anti-VEGF therapy, Vandefonteyne et al. report intravitreal injection of anti-VEGF in 30 out of 82 eyes (36.3%) [10]. This included ranibizumab (11 eyes), aflibercept (3 eyes), and bevacizumab (4 eyes) [10]. The mean number of intravitreal injections was 7.7 with a median of 4 and range from 1 to 32 [10]. The mean duration between diagnosis and first intravitreal injection was 13.6 days [10]. Additional therapies utilized included laser photocoagulation in 22 eyes (26.8%) with a duration between diagnosis and photocoagulation of 4.3 months on average (median 3 months, range 0.25–24 months), photodynamic therapy in 1 eye (1.2%), vitrectomy in 16 eyes (19.5%), and cryotherapy in 5 eyes (6.2%) [10]. The most common indication for vitrectomy was persistent vitreous hemorrhage [10]. This was also the only treatment performed on this cohort in which there was a benefit in visual acuity. The authors postulate that this is due to the vitrectomy clearing the vitreous cavity, while other therapies, namely cryotherapy, laser photocoagulation and intravitreal injections were performed to treat peripheral lesions that were less threatening to the macular region and peripheral enough to not necessarily be associated with a decrease in visual acuity [10]. In a recent study, repeated bevacizumab injections resulted in statistically significant disease resolution on clinical examination, though there was no statistical difference in VA between treated and untreated patients [11]. Lesion ultrasound thickness, the number of disease foci, and number of lesions showed a reduction tendency in this cohort as well [11]. The authors found this to be encouraging, as the treatment group had a more aggressive presenting disease. Additionally, it was thought that the VA of this cohort was affected by coexisting ocular disease, rendering it less relevant for PEHCR monitoring.

In the recent case series by Zicarelli et al., out of 50 eyes and 35 patients, patients either underwent observation, intravitreal injection of anti-VEGF, or photodynamic therapy [8]. Of these patients, 18 eyes (36%) underwent observation, 18 eyes (36%) underwent combined anti-VEGF intravitreal injection with photodynamic therapy, 13 eyes (26%) had intravitreal anti-VEGF only, and 1 eye (2%) had photodynamic therapy only [8]. Authors did not find statistical correlation between the macular involvement of PEHCR and treatment strategy [8]. However, only 4 out of 18 eyes in the observation group developed either macular involvement or vitreous hemorrhage, and the authors did discover a significant inverse correlation between treatment and macular involvement in high-risk eyes [8]. Notably, the risk of posterior involvement of PEHCR lesions was significantly reduced in anti-VEGF treated eyes, advocating for the use of intravitreal anti-VEGF injection in high-risk eyes with vision- or macular-threatening disease [8]. Zicarelli et al. did not treat patients with laser photocoagulation due to a concern that it would not adequately address the underlying choroidal vascular network despite its effectiveness for focal polypoidal dilations [8], although they did treat some patients with photodynamic therapy and combined anti-VEGF with photodynamic therapy. 

The presentation of PEHCR can widely vary from asymptomatic discovery to vision-threatening recurrent vitreous hemorrhage or subretinal fluid and exudate involving the macula. Additionally, the clinical course can be highly variable from instances of spontaneous resolution without impacting visual function with no need for active treatment as evident in our case, to persistent vision decline requiring frequent treatment. Treatment can include anti-VEGF intravitreal injection, photodynamic therapy, or vitrectomy depending on the etiology of vision loss. The peripheral nature of the disease can result in misdiagnosis, though detailed examination with the help of multimodal imaging can improve diagnostic accuracy and distinguish it from intraocular tumors [4,10]. PEHCR lesions are most commonly misdiagnosed or referred as choroidal melanoma [4]. Having the knowledge, experience, and comfort with systematic evaluation of these lesions with appropriate imaging when indicated is essential for safe and value-based care with appropriate observation or escalation of therapy when indicated. Peripheral OCT for PEHCR lesions may allow accurate disease monitoring and active surveillance for treatment indication. As discussed, appropriate treatment plans may significantly vary from observation, as many of these lesions stabilize or regress without intervention, to frequent intravitreal injection of anti-VEGF or vitrectomy, necessary to prevent vision loss due to macular-extending pathology or vitreous hemorrhage [4,10,15]. The widespread adoption of anti-VEGF intravitreal injections has allowed effective treatment options for certain lesion vision-threatening characteristics such as subretinal fluid, although, there is no definitive treatment recommendation for the disease due to the broad spectrum of vision impact, and each case should be individually evaluated and managed.

Further studies of PEHCR are recommended to better characterize the utility of treatment such as anti-VEGF therapies versus observation, ideally with a randomized control trial. While case reports and retrospective reviews exist, there has been no prospective randomized control trial to date. A more extensive dataset with the ability to compare treatment options in addition to a larger-scale comparison of patient demographics, lesion characteristics, anatomical and functional response to treatment, burden of treatment, long-term outcomes and follow-up timelines would allow a better understanding of this disease process and its recommended management. Additionally, further studies of the value of multimodal imaging associated with PEHCR lesions will help guide clinician decision-making not only in treatment indication but also in diagnostic analysis.

## 4. Conclusions

PEHCR is a peripheral vascular disease of the retina that is likely underreported due to its frequently asymptomatic presentation. The etiology has been classified on the spectrum of both AMD and PCV, and while it shares common characteristics with both diseases, PEHCR may represent a separate entity. While vision is often unaffected, a decrease in vision can occur secondary to vitreous hemorrhage or extension of subretinal hemorrhage, fluid, or exudate to the macula. It is important to properly diagnose this disease, as these lesions are commonly referred as concern for choroidal melanomas due to their large, dark, elevated presentation in the periphery. Multimodal testing incorporating FA, a B-scan, and SD-OCT is important for reaching the correct diagnosis. PEHCR can often be observed without treatment, though intravitreal injection of anti-VEGF is increasingly reported to prevent secondary causes of vision loss.

## Data Availability

No new data were created or analyzed in this study. Data sharing is not applicable to this article.
